# Image-Guided Mesenchymal Stem Cell Sodium Iodide Symporter (NIS) Radionuclide Therapy for Glioblastoma

**DOI:** 10.3390/cancers16162892

**Published:** 2024-08-20

**Authors:** Siddharth Shah, Brandon Lucke-Wold

**Affiliations:** Department of Neurosurgery, University of Florida, Gainesville, FL 32608, USA; brandon.lucke-wold@neurosurgery.ufl.edu

**Keywords:** glioblastoma, targeted radionuclide therapy, neurosurgery, brain tumor, cancer

## Abstract

**Simple Summary:**

Glioblastoma (GBM) is the most common glioma, which belongs to the aggressive and malignant type of brain tumor. Conventional treatments such as surgical resection, adjuvant radiotherapy, and concomitant and adjuvant temozolomide chemotherapy have only limited effects. Due to the poor prognosis of patients with GBM, there is an urgent need to research effective new adjuvant treatments. The non-invasive imaging-based detection of glioma stem cells presents an alternate means to monitor the tumor and diagnose and evaluate recurrence. Radionuclide therapy seems promising in the treatment of solid malignant tumors. As for the currently increasing number of studies on NIS radionuclide therapy in vivo and in vitro, we aimed to focus on the therapy in GBM using the effective and feasible image-guided strategy to guide radiotherapy to develop a new clinical therapeutic strategy with societal impact.

**Abstract:**

Background: Glioblastoma (GBM) is a highly aggressive, invasive, and growth factor-independent grade IV glioma. Survival following the diagnosis is generally poor, with a median survival of approximately 15 months, and it is considered the most aggressive and lethal central nervous system tumor. Conventional treatments based on surgery, chemotherapy, and radiation therapy only delay progression, and death is inevitable. Malignant glioma cells are resistant to traditional therapies, potentially due to a subpopulation of glioma stem cells that are invasive and capable of rapid regrowth. Methods: This is a literature review. The systematic retrieval of information was performed on PubMed, Embase, and Google Scholar. Specified keywords were used in PubMed and the articles retrieved were published in peer-reviewed scientific journals and were associated with brain GBM cancer and the sodium iodide symporter (NIS). Additionally, the words ‘radionuclide therapy OR mesenchyma, OR radioiodine OR iodine-131 OR molecular imaging OR gene therapy OR translational imaging OR targeted OR theranostic OR symporter OR virus OR solid tumor OR combined therapy OR pituitary OR plasmid AND glioblastoma OR GBM OR GB OR glioma’ were also used in the appropriate literature databases of PubMed and Google Scholar. A total of 68,244 articles were found in this search on Mesenchymal Stem Cell Sodium Iodide Symporter and GBM. These articles were found till 2024. To study recent advances, a filter was added to include articles only from 2014 to 2024, duplicates were removed, and articles not related to the title were excluded. These came out to be 78 articles. From these, nine were not retrieved and only seven were selected after the removal of keyword mismatched articles. Appropriate studies were isolated, and important information from each of them was understood and entered into a database from which the information was used in this article. Results: As a result of their natural capacity to identify malignancies, MSCs are employed as tumor therapy vehicles. Because MSCs may be transplanted using several methods, they have been proposed as the ideal vehicles for NIS gene transfer. MSCs have been used as a delivery vector for anticancer drugs in many tumor models due to their capacity to move precisely to malignancies. Also, by directly injecting radiolabeled MSCs into malignant tumors, a therapeutic dosage of beta radiation may be deposited, with the added benefit that the tumor would only localize and not spread to the surrounding healthy tissues. Conclusion: The non-invasive imaging-based detection of glioma stem cells presents an alternate means to monitor the tumor and diagnose and evaluate recurrence. The sodium iodide symporter gene is a specific gene in a variety of human thyroid diseases that functions to move iodine into the cell. In recent years, an increasing number of studies related to the sodium iodide symporter gene have been reported in a variety of tumors and as therapeutic vectors for imaging and therapy. Gene therapy and nuclear medicine therapy for GBM provide a new direction. In all the preclinical studies reviewed, image-guided cell therapy led to greater survival benefits and, therefore, has the potential to be translated into techniques in glioblastoma treatment trials.

## 1. Introduction

Glioblastoma (GBM), also known as glioblastoma multiforme, is the most common glioma, which belongs to the aggressive and malignant type of brain tumor. At present, GBM has a low survival rate and effective therapy is limited [1]. Histopathologically, GBM is composed of both the diffuse infiltration zone and the main tumor mass [1]. Conventional treatments such as surgical resection, adjuvant radiotherapy, and concomitant and adjuvant temozolomide chemotherapy have only limited effects [1,2]. The newly adjuvant photodynamic therapy proposed by our team to be combined with temozolomide chemotherapy also has few side effects [2]. However, the recurrence rate and the median survival period of patients with GBM are still significantly worse than other solid malignant tumors [2,3]. Research on using the sodium iodide symporter (NIS) gene to label cancer can open the door for GBM radionuclide theranostics, yet clinical applications are limited due to the limited labeling efficiency [4]. Glioma stem cells express the receptor for the glial cell-derived neurotrophic factor, and mutations can result in the glial cell-derived neurotrophic factor and signal transduction pathways constitutively active in GBM cells [5]. Due to the poor prognosis of patients with GBM, there is an urgent need to research effective new adjuvant treatments [1,5]. The lack of specific target expression in GBM stem cells effectively limits targeted therapy [6]. The non-invasive imaging-based detection of glioma stem cells presents an alternate means to monitor the tumor and diagnose and evaluate recurrence [6]. The sodium iodide symporter gene is a specific gene in a variety of human thyroid diseases that functions to move iodine into the cell [7]. In recent years, an increasing number of studies related to the sodium iodide symporter gene has been reported in a variety of tumors and as therapeutic vectors for imaging and therapy. Gene therapy and nuclear medicine therapy for GBM provide a new direction [7]. Key therapeutic targets of GBM have relatively easily been found and are still being searched for. Radionuclide therapy seems promising in the treatment of solid malignant tumors. As for the currently increasing number of studies on NIS radionuclide therapy in vivo and in vitro, we aimed to focus on the therapy in GBM using the effective and feasible image-guided strategy to guide radiotherapy to develop a new clinical therapeutic strategy with potential societal impact. NIS is a transmembrane glycoprotein found primarily on the basolateral surface of the follicular cells in the thyroid, but also expressed in the parafollicular cells of the thyroid, lactating mammary glands, stomach, and a host of other tissues [8]. It has a highly typical and unique function of concentrating and incorporating iodine, which it usually does by pumping iodide into thyroid cells, where it is then oxidized and incorporated into the precursor molecule of the two thyroid hormones (T3 and T4) [8]. This iodine-concentrating capability of NIS is preserved in well-differentiated thyroid cancers and exploited in the form of radioiodine therapy after pre-treatment with radioactive iodine isotopes, particularly ^131^I, following the resection of the diseased thyroid tissues [8]. It is the radioactivity created by the breakdown of ^131^I that removes the threat of any remaining cancerous thyroid tissues. The competitive inhibitors thiocyanate and perchlorate, as well as the Na^+^Ka^+^-ATPase inhibitor ouabain, can all block NIS-mediated iodide transport [9]. For the past 80 years, radioiodide has been utilized extensively in the treatment of differentiated thyroid cancer. This is due in part to the foundation of functional NIS expression. The “crossfire effect”, which is the radiation of accumulated radioisotopes in NIS-expressing cells on nearby non-expressing cells by particle decay, is linked to the cytoreductive effect of targeted NIS-mediated radioisotope treatment [10].

**Figure 1:**
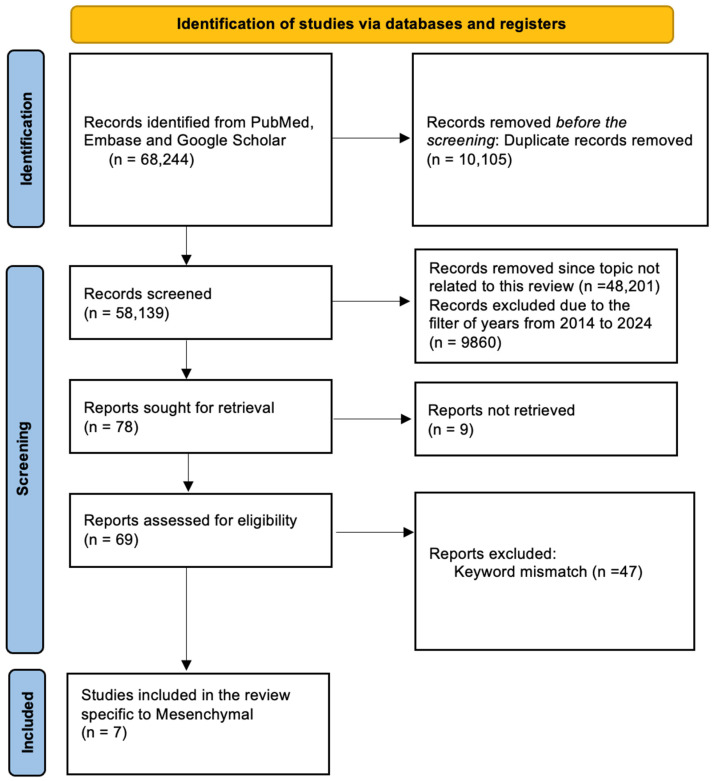
Methodology for systematic selection of articles for this review.

Beyond the application of NIS as a reporter gene for gene therapy with gene-directed enzyme/prodrug cancer therapy, in recent years, there has been great interest in applying NIS as a theranostic tool using radioiodine therapy and in screening systems for the development of NIS-based radionuclide diagnostic and therapeutic strategies [11]. Central to these undertakings, the overall goal is to build up sustaining concentrations of iodide and radioactive iodide that can continue to a level leading to radiation-induced lethal DNA double-strand breaks in the tumor cells [11,12]. With such a powerful built-in negative feedback loop, the function of the iodine concentration mechanism in turning on the expression of the hNIS transgene is necessary to cause tumor cell death [11]. Figure 1 depicts the methodology used to retrieve the articles for this review.

Specified keywords were used in PubMed, Embase, and Google Scholar, and the articles retrieved were published in peer-reviewed scientific journals and were associated with brain GBM cancer and the sodium iodide symporter (NIS). Additionally, the words ‘radionuclide therapy OR mesenchymal OR radioiodine OR iodine-131 OR molecular imaging OR gene therapy OR translational imaging OR targeted OR theranostic OR symporter OR virus OR solid tumor OR combined therapy OR pituitary OR plasmid AND glioblastoma OR GBM OR GB OR glioma’ were also used in the appropriate literature databases of PubMed and Google Scholar. A total of 68,244 articles were found in this search on Mesenchymal Stem Cell Sodium Iodide Symporter and GBM. These articles were found till 2024. To study recent advances, a filter was added to include articles only from 2014 to 2024, duplicates were removed, and articles not related to the title were excluded. These came out to be 78 articles. From these, nine were not retrieved and only seven were selected after the removal of keyword mismatched articles. Appropriate studies were isolated, and important information from each of them was understood and entered into a database from which the information was used in this article.

## 2. Mesenchymal Stem Cells as Carriers of NIS Genes

Mesenchymal stem cells supply bone marrow stroma and have in vitro differentiation capacity into chondrocytes, osteoblasts, and adipocytes [10]. As supporting cells in peripheral tissues, MSCs are closely related to their secretory functions in physiological homeostasis, tissue repair, and fibrosis [11]. Additionally, MSCs exert immunoregulatory effects, improving multiple diseases, such as Graft Versus Host Disease (GVHD) [12]. MSCs have been immediately utilized after transplantation due to their low immunogenicity and the expression of MHC-class-I combination molecules, HLA-G, B7-H1, PD-L1, IDO, and prostaglandin E2, which can inhibit T cell and NK cell responses, particularly MHC-class-II negative peripheral blood MSCs, which can induce immune tolerance similar to fetal MSCs [12]. However, these molecules and cytokine-mediated immunoregulatory functions are unstable and are easily influenced by the cell microenvironment, such as inflammation and hypoxia [13]. Prior research has found that affected tissues release more IL-1β, IL-6, and TNF-α when MSCs are transplanted. MSCs have a strong homing ability for inflammatory tissues and have an abundant blood supply and rich nutrient sources [13]. After 6 weeks of injection, a small number of MSCs are undifferentiated and can be detected in the lungs, kidneys, and liver. However, they can only be detected by detection techniques, such as luciferase, PCR, qPCR, lentivirus-GFP, and cyclophosphamide-GFP, and fewer studies have used animal models of heart disease specifically [14]. In addition, MSCs possess a large, flat, spindle-shaped morphology, representing an adherence-independent characteristic of the suspension culture, and are not sensitive to serum-containing medium or carrier contact. After a certain period of exponential growth, MSCs may enter the G0 phase, exhibiting cell senescence, cell aging, cell death, and G1/S blocking, thereby negatively impacting proliferation [15]. Finally, MSCs are recognized as the nuclei and cells by karyotyping techniques, which change the chromosomal number and affect cell differentiation potential, the secretion of cytokines, biological characteristics, and tumorigenicity [16].

Bone marrow-derived mesenchymal stem cells and umbilical cord blood-derived mesenchymal stem cells are the only stem cell types that can be banked following plastic adherence and long-term expansion [15]. Mesenchymal stem cells systematically inhibit T and B cells while expressing functional NIS, but are thereafter largely replaced by functionally competent embryonic stem cell-derived mesenchymal stem cells [16]. Co-transduction of NIS and thyroid peroxidase (TPO) for the non-immunogenic target-specific NIS-probe is another method [16]. Specifically, mesenchymal stem cell attachment onto NIS-expressing primary non-thyroid cells may be the easiest if the NIS expression level is checked before the initial stem cell isolation [16]. The potency of heterologous or allogeneic NIS-mesenchymal stem cells defined here should stimulate further in vivo NIS/gene delivery model development. In 2003, Herzog et al. proved that BM-derived MSCs could migrate to damaged brain tissues and transform into neurons, astrocytes, and oligodendrocytes [17]. The observation showed that MSCs participated in neurodegenerative disease treatments. The BM-derived MSCs could also cure chronic spinal cord injury in mice. Intravenous transplantation of MSCs reduced astrogliosis, induced oligodendrocyte synthesis, and promoted serotonergic axon regeneration [17]. In 2003, Angelopoulou et al. investigated the properties of BM-derived MSCs for expressing SP during adipogenic differentiation [18]. This finding identifies the direct action of MSCs to angiogenesis. Furthermore, BM-derived MSCs take part in the skeletal muscle-repairing process. After muscle injury in mice, the injected BM-derived MSCs repair the muscle and downregulate the migration inhibitory factor. MSCs could also participate in the recovery from lung injury [19]. They created an antifibrogenic effect and restored the proliferation of alveolar type II cells. Mesenchymal stem cells (MSCs) are self-renewing and pluripotent, and can differentiate into tissues like adipocytes, chondrocytes, osteoblasts, etc. Many kinds of MSCs could express normal tissue-specific proteins when seeded or transplanted in some kinds of tissues. Umbilical cord blood-derived MSCs are CD34-negative, CD45-negative, and HLA-DR-negative. Human umbilical cord blood-derived MSCs could differentiate into cells expressing liver-function proteins [20]. Human placental- or umbilical cord blood-derived MSCs are suitable for obtaining CB, and the process of collecting CB samples is non-invasive. It avoids donor morbidity and infection risks so that the safety issue is reduced. Moreover, it has little HLA identity, so transplantation of embryonic stem cells (ESCs) or MSCs from CB may reduce the side effects of immune rejection [21]. The characteristics of low immunogenicity and potent immunosuppression make MSCs suppress immune rejection, solve engraftment difficulties, and ameliorate the stem cell niche environment. Remarkably, the umbilical cord blood-derived MSCs sustain high paracrine activity with high inflammatory cytokine levels. Cell damage and cryopreservation strategies impact paracrine activity [22].

### 2.1. Origin and Differentiation Potential

MSCs are an adult and multipotent non-hematopoietic cell type. MSCs can differentiate across the mesodermal lineages, i.e., chondroblasts, adipogenic, osteoblastic, myoblastic, and osteoblastic lineages, and give rise to other connective tissue cells, i.e., fibroblasts, endothelial, and epidermal cells [23]. Additionally, MSCs differ from other stem cell types in that they are relatively easily procured from adults, induced to proliferate in culture, and employed in cell-based bioengineering strategies. These unique features have provided strong rationales for extensive empirical investigations and applications, and there has been a concomitant explosive interest in the cellular and molecular biology of MSCs en route to understanding their fundamental properties and designing useful translational strategies. MSCs that can undergo adipocyte, chondrocyte, osteoblast, and other lineage differentiation were initially isolated from rodent bone marrow by Friedenstein [24]. However, MSCs exist not just in the bone marrow; they are scattered throughout various tissues, such as the subcutaneous tissue, dental pulp, kidney, amniotic fluid, cord blood, and adipose tissue. Among these resident MSC populations, adipose MSCs (ASCs) and bone marrow MSCs (BMSCs) have been intensively identified for their multi-lineage differentiation and powerful homeostasis [25,26]. Especially, ASCs are easier to obtain and their source is abundant in comparison with BMSCs, thus they are potential MSCs in medicine and research. Therefore, mesenchymal stem cells are considered to be effective target cells for the deep therapy of various diseases.

### 2.2. Immunomodulatory Properties

Human mesenchymal stem cells (hMSCs) are self-renewing progenitors best known for differentiation into mesenchymal lineage cells such as osteocytes, adipocytes, and chondrocytes. hMSCs can also differentiate into endodermic-like cells, for example, hepatocytes, and neurogenic-like cells, for example, neurons and glia; these cells can participate in tissue repair and regeneration mainly through paracrine effects. The cells surrounding the MSC and the microenvironment in which they are located significantly affect their potential role in the immune response [27]. Endogenous MSCs are found in bone marrow, pericytes from various tissues, fibroblasts, and neural cells. Due to the low number of MSCs in human tissues, explantation techniques are used to expand these cells in in vitro cultures. In clinical trials, MSCs are safe and hold particular promise for their immunophysiological properties in the treatment of various diseases [28].

MSC-associated features include their apparent exclusion from the different types of lymphoid and myeloid cells; they are immunosuppressive. These properties, together with the property of suppressing the proliferation of peripheral blood lymphocytes that do not produce lymphokines, enable them to absorb or modulate immune responses [29]. Although these effects have been extensively studied in a large number of in vitro allogeneic and xenogeneic studies, MSC actions in vivo are less defined. In particular, labeling, ex vivo expansion, and reimplantation of MSCs showed that, a few days later, they were rejected in a reaction mediated by T cells or NK cells. Lower immunosuppressive impacts in vivo were observed, although one working mechanism of MSC action includes their participation in the suppression of lymphopoiesis in the bone marrow [30]. Control of lymphopoiesis is achieved through the withdrawal of IL-7 and CXCL12 support from the stem/progenitor cells. MSCs lack HLA class II expression. Even in conditions in which major histocompatibility complex class I expression was enhanced, few NK-activating molecules of MSCs were observed [31]. Most of the evidence is consistent with an immune escape mechanism. MSC immunosuppression was effective either in the presence or absence of a descending number of specific patient lymphoid cells. Furthermore, the suppressive effects of patient hMSCs were maintained in peripheral blood lymphocyte samples harvested after transplant, suggesting resistance to tolerance factors [32,33]. The mechanism(s) that MSCs use to suppress immune responses is complex but mainly involves localized effects via the release of soluble factors. The immunosuppressive pattern of MSCs was also involved in the selective accumulation of these cells in some tumors [34]. Okolicsanyi and Griffiths assessed the isolation, expansion, and differentiation of WJ-MSCs and U-CMSCs isolated from different gestational ages of Wharton’s jelly (WJ) into osteoblasts, adipocytes, and neural progenitor cells and the hMSC cell surface markers, revealing that WJ-MSCs may be a potential stem cell therapy source for nervous system injury and that the immunophenotypes of the expanded isolated U-CMSCs did not change with in vitro culturing [35]. These cells maintained an extraordinary growth rate, while the WJ-MSCs maintained their structural and functional features. Balakrishnan assessed the immunomodulatory properties of different sources of hMSCs, ranked the comparison based on the intensity of expression of immunomodulatory and cytokine receptors, and concluded that WJMSCs demonstrate intense anti-inflammatory and anti-apoptotic mechanisms, such as the release of immunoregulatory and pro-angiogenic factors [36]. In summary, the results presented by Balakrishnan and Okolicsanyi and Griffiths provide evidence for the potential role of umbilical cords in the use of U-CMSCs for immune system regulation in different disorders.

### 2.3. Tumor-Tropic Migration

MSCs have the unique ability to migrate towards tumors. Studies have determined the migration ability of both mouse MSCs and human MSCs towards a variety of tumor types, both in vitro and in vivo [33]. As a consequence, MSCs were found to localize and engraft in various tumor models following their transplantation into animals. Interestingly, MSCs possess the ability to specifically migrate towards sites of sterile inflammation and ischemia and to migrate towards sites of allogeneic hematopoietic stem cell transplantation [37,38]. Although the natural physiological function of MSCs to migrate towards sites of injury and inflammation is beneficial in tissue repair, it has been suggested that MSCs could potentially enhance tumor metastasis through their migration tropism [39]. Some groups hypothesized that MSCs have anti-tumor abilities based on evidence showing that some cytokine-activated MSCs can inhibit the growth of various tumor-type cells in vitro [40]. The filing of MSCs with some cytokines, such as interferon-c (IFN-c), can enhance the in vitro anti-tumor ability of MSCs. In addition, results from several in vivo studies clearly show that MSCs can inhibit tumor growth if they are co-administered with appropriate cytokines such as GM-CSF or IFN-c [41]. There are much controversial data on the interaction between MSCs and tumor cells: MSCs can enhance or inhibit tumor cell invasiveness in the presence or absence of tumor necrosis factor-a (TNF-a). The existing knowledge about the interaction between MSCs and different types of tumor cells implies that the design of MSC-based gene delivery as an “effector” therapy against systemic malignant tumors should take into account not only the tumor-tropism of MSCs, but also the effect of the tumor on the MSCs themselves, in particular, how MSCs are activated, reprogrammed or get sick when they meet tumor cells, and how these phenomena would synergistically impact on the ultimate gene therapy efficacy [42]. Due to their innate ability to locate tumors, mesenchymal stem cells (MSC) are used as vehicles for tumor treatment. Growth factors, chemokines, and inflammatory cytokines are all produced at higher levels in tumors, which encourages MSCs to actively recruit into the tumor microenvironment and aid in the formation of the tumor stroma [43]. Because MSCs are easily extracted, amplified, and transplanted across the allogenic barrier, they are highly suited for therapeutic uses. MSCs that have undergone genetic engineering hold promise as delivery systems for therapeutic genes like NIS. In early-stage human clinical studies, the use of modified MSCs to treat solid tumors is currently being investigated [44]. Preclinical research utilizing xenograft tumor mouse models has shown the potential of CMV (cytomegalovirus) promoter-driven MSC-mediated NIS gene delivery, with successful selective NIS expression in tumors and metastases as well as a strong therapeutic response following [^131^I]NaI application [45]. MSC engineering has been exploited for cell-mediated gene therapy and for carrying therapeutic transgenes that protect the engrafting cells from the conditioning cytotoxic insult or that reinforce the functions of the stem/progenitor cells in the host recipients, promoting their engraftment, the differentiation processes, or conditioning a permissive local environment [46]. However, the beneficial effects have been obtained only in a subset of the genetically manipulated cells, and the strategies available are still very expensive and potentially associated with risk factors. Some laboratories have reported results with a more efficient MSC transduction, alongside the use of different transduction systems such as commercial viral vectors or the production of viral vectors derived from in-house developed stable packaging cells [47,48]. Radioisotope therapy can be successfully carried out with cells expressing the sodium iodide symporter (NIS). Mesenchymal stem cells (MSCs) have been put forward as desirable vehicles for NIS gene transfer since they can be transplanted in a variety of different ways [49]. They can contribute to viral clearing in in vivo experiments and do not replicate in the target tissue, therefore providing tumor-specific pNIS expression [50]. This latter property can significantly reduce the potential toxicity when the NIS-targeted malignant cells undergo efficient radio uptake by the gamma emitter radionuclides.

## 3. Mechanism of NIS Gene Delivery

A large number of gene-transfected human MSCs could transmigrate into the intracranial lesions expressing the transferred NIS under cytokine enhancement, and the NIS-expressing cells could effectively concentrate radioiodine, therefore causing the accumulation of activity in vivo [51]. Moreover, the in vivo data of high retention time and high tumor-to-normal tissue ratio indicate the technique specificity and therapeutic potential. The manipulable MSC pool easily obtained from humans is capable of reliably carrying and expressing the target gene, thus providing a feasible way for adoptive tumor radiotherapy [52,53]. Human umbilical cord blood (hUCB)- and human bone marrow (hBM)-derived mesenchymal stem cells (MSCs) were effectively transfected with a plasmid vector expressing the marker sodium iodide symporter (NIS) using the RRL-mediated gene transfer technique [54,55]. The gene-transfected MSCs were capable of migrating to the basal ganglia of the infants. The NIS on the membrane could extract iodide from the media and then concentrate radioiodine in the cells [56,57]. Data showed that large numbers of administered gene-transfected human BM-MSCs could transmigrate into the intracranial lesions and express the transferred human NIS gene under stimulations of granulocyte–macrophage colony-stimulating factor (GM-CSF) or endostatin [58,59]. The labeled MSCs distributed in the basal ganglia of the brains were assayed in the lymphoma-bearing mice by single photon emission computed tomography [60]. 

### Advantages and Limitations

Multiple potential advantages have been proposed for the use of MSCs to deliver NIS. First, MSCs can be readily isolated from a patient’s bone marrow and expanded under GMP conditions, thus providing an autologous, relatively inexpensive source of cells that are well tolerated in most patients [61]. Second, the migratory and tropic properties of MSCs likely contribute to MSC distribution in areas of injury, including various premetastatic niches, and facilitate MSC extravasation through basement membranes and endothelial cell tight junctions to engraft into the target tissue, particularly following tissue-specific damage [62]. Despite the advantages of utilizing MSCs as a gene delivery vehicle, several important considerations also need to be addressed. Direct injection of radiolabeled MSCs into malignant tumors could reduce tumor burden by depositing what would be a therapeutic dose of beta radiation with the added advantage of localizing only in tumors, not in normal tissues [63]. Direct injection of radiolabeled MSCs into glioblastomas could potentially address the challenge posed by the tumor’s multicentric nature, which involves the presence of multiple, spatially distinct tumor sites within the brain. MSCs have a natural tropism for tumors, meaning they can migrate towards and localize at tumor sites, including those that are not easily accessible. By radiolabeling these cells, the therapy can deliver targeted radiation directly to the tumor sites, regardless of their location, thereby addressing the primary and secondary tumor foci. This targeted approach helps in concentrating the therapeutic effects on the tumor cells while sparing surrounding healthy brain tissue, offering a more effective treatment for the multifocal nature of glioblastomas. At the recent NRVS Congress, an abstract by Lee et al. presented phase I data using directly injected Ad-TC-M1-MSCs into patients with metastatic breast cancer lesions and reported both safety and patient-derived pain reduction [64]. MSCs have been found effective for several diseases in phase I/II clinical studies but have yet to show a convincingly large effect when tested in additional patients in Phase III trials [65]. There are several obstacles to stem cells themselves that need to be overcome, such as the inability to drive differentiation into the appropriate lineage, poor survival of the implanted cells, and the inability of the implanted cells to cooperatively function on a large scale due to non-repair cells [65]. In cancer treatment using stem cells as gene delivery vehicles, additional obstacles prevent successful treatments. Since tumors are heterogeneous, it is unlikely that a uniformly radiolabeled MSC population can uniformly target a solid mass [66]. Furthermore, MSCs are part of the immunosuppressive environment of the tumor that generates them, and efforts to use transformed MSCs that are not immunomodulatory can improve cell survival and the effect of the MSCs. The application of endogenous MSCs can avoid an immunological response to allogeneic antigens [67]. Many cancer entities are MSC-homing, and MSCs have been demonstrated to be capable of carrying the NIS-transgene gene to a primary or metastatic cancer site to synthesize the NIS protein, which can trigger cancer cell death. MSCs also work as a stromal component that can resist forming radiation-induced immune cell populations and abrogating immune-based tumor rejection in postponed tats [68]. Although all of these characteristics make MSCs an optimal gene delivery vehicle, there are still some disadvantages. First, a suitable method for MSC transplantation and the timing of NIS-MSC transplantation for a better outcome have not been determined. Second, the final destination of NIS-MSCs and the dynamic changes at the molecular level of the target tumor-expressing NIS have not been analyzed [69]. Third, the probable tumor-forming risk resulting from the interaction between MSCs and the activated host’s microenvironment also remains unclear. In addition, MSCs can also be dedifferentiated to regain tumorigenic potential after being modified by the NIS gene, which can limit the applicability of MSCs as a gene delivery vehicle. Somatic cell dedifferentiation is concerning for future applications, in particular when the cell sources for reprogramming are used as carriers for gene therapy [70]. Taken together, based on these findings, we foresee that a combination therapy approach will be required to achieve effective antitumor therapy with the NIS gene to treat cancer.

**Figure 2:**
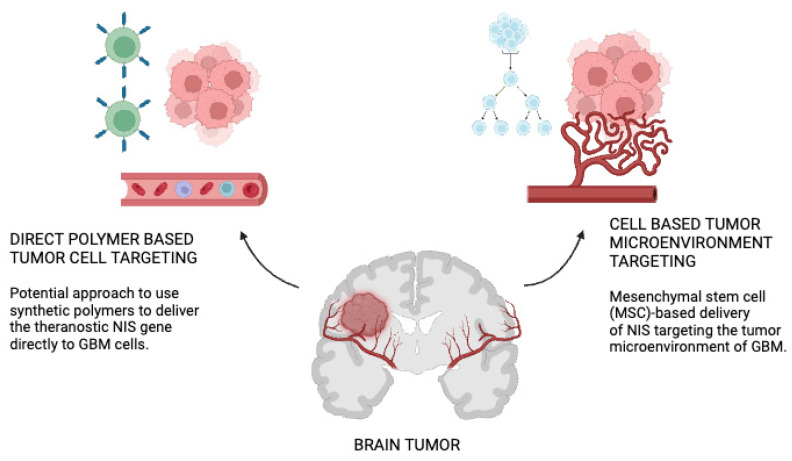
NIS gene delivery strategies to glioblastoma.

NIS gene delivery strategies to GBM have been depicted in Figure 2. On the left-hand side is a potential approach to use synthetic polymers to deliver the theranostic NIS gene directly to GBM cells. The polymer backbone is functionalized with ligands (targeting domain) that have a high affinity to cell surface receptors that are overexpressed in GBM cells. Polymers are loaded with NIS pDNA. Following systemic administration of polymers, the DNA is released to the GBM cells after binding of the polymer to the cell receptor [71]. On the right-hand side is the mesenchymal stem cell (MSC)-based delivery of NIS targeting the tumor microenvironment of GBM. MSCs can be easily isolated from patients from different tissue sources (e.g., bone marrow or adipose tissue) and genetically modified with the NIS gene under the control of tumor-stroma-specific gene promoters. Engineered MSCs can be amplified in the laboratory and systemically administered back to the patient or over the allogenic barrier [72]. Tumor-secreted factors (e.g., inflammatory cytokines) promote direct migration and extravasation of MSCs to the GBM where they become part of the tumor stroma. NIS expression is induced after promoter activation. Following successful NIS gene transfer using both delivery platforms, diagnostic and therapeutic applications of radioactive NIS substrates can be applied. pDNA; plasmid DNA [72]. Table 1 compares these two techniques. The reason for us to choose MSCs was because MSCs naturally adhere to tumors, which may improve the efficacy and specificity of NIS administration. By acting as carriers for NIS and other therapeutic substances, MSCs provide a multimodal approach to the therapy of tumors. Using MSCs to distribute NIS is a fresh and exciting tactic that has the potential to get around some of the drawbacks of conventional polymer-based systems. The therapeutic potential of MSCs is gaining attention, which makes this a pertinent and current field of study. Targeted cancer therapy may be more feasible and scalable with the application of MSCs, which may translate more easily into clinical settings.

## 4. Image-Guided Approaches in Radionuclide Therapy

The NIS, under the control of a modifying and specific promoter, can guide an enhanced and safer clinical translation potential with the capability for real-time non-invasive imaging that helps evaluate drug delivery, allowing for optimization and might even direct personalized therapy [73]. The use of the herpes simplex virus type 1 thymidine kinase (HSV1-tk) as reporter genes for PET imaging has been reported in preclinical models as a potential for the selective and non-invasive evaluation of genetic immunotherapy for GBM [73]. Magnetic resonance imaging and other more advanced imaging techniques have also been explored to extract other information from NIS expression and gene therapy response in GBM as compared to NIS radionuclide therapy [74]. Each method in the literature has indications and limitations; the choice should rely on the resources available and the specific questions addressed.

NIS is a potent theranostic gene that, as the preceding sections have shown, enables effective molecular therapy monitoring following radionuclide administration [74]. NIS possesses several attributes of an ideal reporter gene as well: This protein is found spontaneously in thyroid follicular cells and is neither immunogenic nor cytotoxic to cells. Since iodide buildup may only take place in active cells, cell viability is linked to functional NIS activity. The signal is concentrated due to the buildup of radiolabeled substrates caused by the active transport of substrates [75]. As a result, compared to a reporter that only binds its substrate stoichiometrically, the detection sensitivity is greater. Because NIS translocates different substrates, it may be used to localize NIS-positive cells using a variety of conventional nuclear medicine imaging methods. Gamma scintigraphy is a diagnostic technique that uses gamma rays to create images of the internal structure and function of organs [75]. Single-photon emission computed tomography (SPECT) is an advanced form of gamma scintigraphy that provides three-dimensional images. In SPECT, the gamma camera rotates around the patient, capturing multiple two-dimensional images from different angles [76]. Gamma scintigraphy and SPECT are made easier by the active transport of ^123^I, ^125^I, ^131^I, ^99^mTc, and ^188^Re. These are used in diagnostic imaging, research, laboratory settings, therapeutic purposes, and nuclear medicine for a variety of imaging studies, including bone scans, cardiac imaging, and renal scans [76]. Furthermore, due to their beta decay, ^131^I and ^188^Re are useful radionuclides for medicinal purposes [76]. In clinical settings, planar scintigraphy or SPECT have been the primary tools for molecular imaging of NIS. On the other hand, PET imaging of functional NIS expression offers the possibility of enhanced sensitivity, resolution, and efficient quantitative analysis [76]. The most well-known and often used positron emitter for NIS-mediated PET imaging in preclinical and clinical settings is ^124^I [77]. Recently, a new tracer for NIS-based PET imaging was developed. Due to the radiochemical and physical characteristics of 18F, 18F-Tetrafluoroborate (TFB) has been presented as a possible replacement for ^124^I. TFB offers several benefits for regular diagnostic use. Because of its shorter half-life (110 min vs. 100 h), branching ratio (97 vs. 23%), and, most importantly, its lower positron energy (Emax; 0.634 vs. 2.14 MeV), TFB is superior to ^124^I in terms of producing PET images that are crisper and less blurred [78].

GBM is the most aggressive brain tumor in humans, and its current treatment strategies fall short of survival benefits. However, the recent advancements in molecular imaging have allowed for improvements in the disease’s initial diagnosis and monitoring. The sodium iodide symporter (NIS) has been studied as a reporter gene to express NIS protein in GBM cells [79]. This results in NIS-expressing GBM becoming a target for radionuclide therapy using beta-emitting radionuclides [80]. These radioisotopes, such as 131 iodine, have been shown to reduce GBM growth in preclinical and early clinical studies. But, maintaining an ideal drug delivery and therapeutic effect using radionuclides in humans, and not only targeting NIS-expressing GBM cells but also the surrounding cells in the GBM microenvironment, needs to be targetable with imaging or treatment [81].

## 5. Image-Guided NIS Radionuclide Therapy in Glioblastoma

Image-guided radionuclide therapy (RNT) using the sodium iodide symporter (NIS) has demonstrated potential in preclinical animal models of GBM. However, there are only limited studies on its use in veterinary and human cancer patients to date. This narrative review considers the published work on NIS RNT in GBM cases and hopes to guide future investigator-initiated, and hopefully some multicentered clinical, studies [82]. These studies utilized more conventional intraoperative image-guided NIS-targeted RNT than in other solid tumors due to the logistical difficulties of radiation safety regulations and limitations in the NIS RNT agents applied, with concomitant radiation damage to normal brain tissue [83]. Novel therapeutic options for GBM are desperately needed, as it is a very complicated tumor that uses several pathways to avoid therapy [83]. Due to its ability to block gene vectors and radiotracers, the blood–brain barrier (BBB) is one factor limiting the effectiveness of GBM therapy and detection [83]. The small-sized radionuclides employed in NIS-mediated radionuclide imaging and treatment can diffuse into the tumor and pass through the blood–brain barrier without the need for complicated radiolabeling processes [84]. Numerous preclinical investigations have exhibited the possible utility of NIS in glioma imaging and treatment [84]. A rat model with intracerebral F98 gliomas that had been retrovirally transduced with human NIS was utilized in research by Cho et al. The scientists demonstrated the potential for non-invasive glioma imaging using [^99^mTc]pertechnetate- and [^123I^]NaI-scintigraphy, and they also demonstrated that rats receiving ^131^I treatment had longer survival times [85]. When the human glioma cell line U87 was injected into the right armpit of mice with xenografted tumors, it was transfected with a recombinant lentiviral vector carrying human NIS. Guo et al. [86] then reported imaging and therapeutic research using ^188^Re and ^131^I. According to gamma camera imaging, in vivo imaging data demonstrated ^188^Re/^131^I accumulation in the NIS-containing tumors. Mice treated with ^188^Re or ^131^I saw an effective decrease in tumor volume when compared to untreated control mice [86]. In another study, Opyrchal et al. [87] reported effective [^123^I]NaI or [^99^mTc]pertechnetate gamma camera or microSPECT/CT imaging of s.c. and orthotopic murine GBM xenografts following intratumoral infection with the measles virus encoding NIS (MV-NIS) to induce NIS expression in brain tumor tissue. This study used one of the most extensively explored oncolytic viruses for NIS gene transfer [87]. In both glioma scenarios, combined radiovirotherapy with MV-NIS and ^131^I produced better anticancer activity and survival than virotherapy alone.

With MSCs designed to trigger NIS expression in response to IL-6 promoter activation, a novel tumor-targeted gene therapy strategy for GBM may be possible. This was studied by Kitzberger et al. [88] who found that when IL-6-NIS-MSCs were applied to tumors, there was an increase in radiotracer uptake by 18F-Tetrafluoroborate-PET/magnetic resonance imaging (MRI) as compared to animals who received wild-type MSCs. NIS protein expression in cancers was observed by ex vivo investigation of malignancies and non-target tissues [88]. Following IL-6-NIS-MSC administration, ^131^I treatment dramatically slowed tumor development as measured by MRI and increased the median survival of GBM-bearing mice to 60% as compared to controls. In another recent study, they created dual-targeted NIS plasmid DNA complexes with targeting ligands for the EGFR and transferrin receptor (TfR), offering the possibility of active transport across the BBB and subsequent targeting of tumor cells [88]. TfR- and EGFR-dependent transfection efficiency as well as NIS-specific iodide absorption of dual-targeted polyplexes were validated by in vitro^125^I transfection assays [89]. Employing 18F-labeled tetrafluoroborate (TFB) as a tracer, positron emission tomography (PET) imaging was used 48 h after intravenous polyplex injection to evaluate in vivo gene transfer in mice harboring orthotopic U87 GBM xenografts [89]. In comparison to animals treated with EGFR-mono-targeted polyplexes (0.33% ± 0.03% ID/mL) or TfR-mono-targeted polyplexes (0.27% ± 0.04% ID/mL), the tumoral 18F-TFB uptake of mice treated with dual-targeted polyplexes (0.56% ± 0.08% ID/mL) was considerably greater [90]. The application of ^131^I produced a better therapeutic impact of the dual-targeted therapy in therapy experiments, as evidenced by a notable delay in tumor development and an extended survival time [90]. Table 2 summarizes the main articles chosen for this review article.

## 6. Challenges and Future Directions: Discussion on Image-Guided NIS Radionuclide Therapy for Glioblastoma

In NIS gene therapy, the malignant cell should only acquire NIS expression that is both restricted to the tumor microenvironment compartment and configured to incorporate and concentrate the radioactive substrates, namely, radioiodine or other NIS substrates [8]. Only the self-destruct property for this focalized area of NIS gene modification is desirable. Lastly, the reduction in radiation exposure level and delaying long-term radiation effect on the remaining normal brain tissue constituent’s health by the NIS substrate after GBM is considered [10]. The following challenges limiting the clinical development of NIS radionuclide therapy for GBM are discussed. Image-guided NIS radionuclide therapy for GBM is challenged by several factors herein summarized: lack of tumor-specific target, unwanted gene transfer to normal brain, confined gene expression within the tumor, specific overexpression level for NIS, the direct relationship of uptake level and efficacy, a minimized detrimental effect on the patient, and dose planning and prediction of therapeutic effect [74]. In these settings, the novel tumor-targeting principles for NIS radionuclide therapy are crucial [87]. The most encouraging outcomes in the diagnostic imaging series came after a single intravenous MSC treatment followed by the delivery of radioiodide 48 h later. The maximum radioiodide uptake, the efflux from the tumor setting, and the average biological half-life within the tumor environment were found to be higher when compared to the administration of radioiodide 72 h after single or multiple MSC injections, as determined by ^124^I-PET imaging. These findings led to an increased calculated tumor-absorbed dose for ^131^I. The in vivo results and the ex vivo study of NIS expression showed a greater number of NIS-positive cells 48 h following a single MSC treatment as opposed to 72 h following a total of three MSC injections [91]. 

Most preclinical studies have shown great potential for the MSCs engineered to express the theranostic NIS gene as an anticancer agent for the treatment of GBM.

## 7. Conclusions

While image-guided NIS radionuclide therapy has the potential to improve GBM treatment, several steps need to be addressed to translate the preclinical results into meaningful clinical practice. In all the studies, invasion using the ablation technique was carried out immediately before the administration of both NIS-hAMSC and ^131^I. The schedule does not reflect the actual benchmarks of patient care. Moreover, clinical outcomes in patients cannot be judged based on the metastasis characteristics of mouse models. They were supposed to eat before ^131^I administration in the report, but the actual treatment may not be sustained when translated to the clinical application. The duration and intensity of the treatment need further optimization, and the present study cannot completely replace pre-existing therapy methods. This marks a foundation for adjuvant or palliative therapy for poor prognosis gliomas, especially in elderly patients. With the continuous development of image-guided NIS radionuclide therapy, better efficacy can be achieved, and frontal lobe function damage can be avoided or minimized. The prevalence of GBM and the absence of standard effective treatments for the condition have contributed to research on possible treatment breakthroughs, among them the sodium ion symporter radionuclide therapy. In this narrative literature review, the process of opening the blood–brain barrier to establish T cell recruitment to the GBM niche before the administration of anti-PD-1 has the potential to provide a more efficient protocol for GBM treatment, especially with the development of image-guided NIS radionuclide therapy [98]. In all the preclinical studies reviewed, image-guided cell therapy led to greater survival benefits and, therefore, has the potential to be translated into techniques in glioblastoma treatment trials.

## Figures and Tables

**Table 1:** Advantages and Disadvantages of Polymer-based and MSC-based Delivery Strategies.

NIS Gene Delivery Strategies	Polymer-Based	MSC-Based NIS Delivery
Advantages [71,72]	Specificity: Polymers can be engineered to target specific tumor cells, minimizing off-target effects. Controlled Release: Polymers can be designed for controlled release of therapeutic agents, ensuring sustained drug availability at the tumor site. Versatility: A wide range of polymers can be used, allowing for customization based on the drug and tumor type. Reduced Immune Response: Some polymers are biocompatible and less likely to trigger an immune response.	Tumor Homing: MSCs have an inherent ability to home to tumor sites, enhancing targeted delivery. Versatile Carrier: MSCs can carry multiple therapeutic agents, including NIS for targeted radiotherapy. Minimal Invasiveness: MSCs can be administered systemically, reducing the need for invasive procedures. Reduced Immunogenicity: MSCs are less likely to provoke an immune response, especially if derived from the patient.
Disadvantages [71,72]	Limited Penetration: Polymer–drug conjugates may have limited ability to penetrate deep into tumor tissues. Complex Synthesis: The design and synthesis of polymer–drug conjugates can be complex and costly. Potential Toxicity: Some polymers may exhibit toxicity or cause adverse reactions. Clearance: Polymers can have varied clearance rates from the body, affecting the duration and effectiveness of the therapy.	Variable Homing Efficiency: The efficiency of MSC homing to tumor sites can vary based on tumor type and microenvironment. Safety Concerns: There are concerns about the potential for MSCs to promote tumor growth or metastasis. Complex Handling: MSCs require careful handling and culture, which can be technically demanding. Regulatory Hurdles: The use of MSCs in therapy faces significant regulatory challenges.

**Table 2:** Articles on NIS and mesenchymal stem cells in glioblastoma therapy.

Studies	Abstract	Conclusion
Mesenchymal stem cell-mediated image-guided sodium iodide symporter (NIS) gene therapy improves survival of glioblastoma-bearing mice [91]	Due in part to their innate capacity to locate tumors, mesenchymal stem cells (MSC) have become viable cellular carriers for the delivery of therapeutic genes in cancer treatment. The sodium iodide symporter (NIS), a theranostic gene, is a promising target for non-invasive radionuclide-based imaging and therapeutics. In this work, we used genetically modified MSCs to target the NIS gene for tumor-targeted transfer in experimental glioblastoma (GBM), a tumor with a very bad prognosis.	By using NIS-mediated in vivo imaging, a strong tumoral NIS-specific radionuclide accumulation was seen following the administration of NIS-MSC and radioiodide. Tumor-selective MSC homing was seen in conjunction with NIS expression in GBM and non-target tissues stained with NIS immunofluorescence. When compared to controls, the application of therapeutically effective ^131^I resulted in a considerable delay in tumor development and an extended median survival following NIS-MSC therapy.
Bone marrow-derived mesenchymal stem cell-mediated dual-gene therapy for glioblastoma [92]	A highly effective BMSC-based therapy approach has been created that permits the concurrent elimination of implanted BMSCs following glioblastoma treatment, evaluation, and suppression of tumor angiogenesis. The human sodium iodide symporter (NIS), which is involved in the uptake of radioisotopes and is controlled by the early growth response factor 1 (Egr1), a radiation-activated promoter, and the angiogenesis inhibitor kringle 5 (K5) of human plasminogen were engineered to co-express in BMSCs.	Mesenchymal stem cells (MSCs) have been used as a delivery vector for anticancer drugs in many tumor models due to their capacity to move precisely to malignancies. Tumor necrosis factor apoptosis-inducing ligand, or NIS, has been engineered to express and deliver immunomodulatory cytokines such as interleukin-12, interferons (IFN) like IFN-α and IFN-β, prodrug converting enzymes like thymidine kinase from the herpes simplex virus, and many other tumor types. The administration of ^131^I after systemic MSC-mediated NIS gene transfer caused a notable postponement in the formation of tumors. K5 and NIS were selected as the treatment agents in this investigation because of their strong synergistic anticancer impact.
The sodium iodide symporter (NIS) as a theranostic gene: its emerging role in new imaging modalities and non-viral gene therapy [93]	The sodium iodide symporter (NIS) was cloned in 1996, opening the door to its potential application as a potent theranostic transgene. Innovative gene therapy approaches use therapeutic radionuclides after image-guided selective NIS gene transfer in non-thyroidal cancers. The development of the NIS gene therapy approach, which uses genetically modified mesenchymal stem cells and synthetic polyplexes as selective non-viral gene delivery vehicles, has advanced significantly over the previous 20 years, as this overview demonstrates.	The tumor micromilieu may potentially be involved in the control of NIS function and/or NIS membrane targeting, which might impact the effectiveness of NIS gene therapy techniques. Extensive evidence from advanced cancer models, including our own data, suggest that the NIS gene therapy idea may be extended to low volume, disseminated illnesses like glioblastoma. NIS transgene expression can be comparatively low in low volume diseases. In this case, the great sensitivity and resolution of emerging imaging techniques should be quite helpful in tailoring treatment plans.
Iodine 125-labeled mesenchymal–epithelial transition factor binding peptide-click-cRGDyk heterodimer for glioma imaging [94]	Using mini polyethylene glycol-conjugated cMBP-3 glycine (GGG), a single name of amino acids (SC) (Ser-Cys), and cRGDyk through a click (1° + 3° cycloaddition), a cMBP-click-cRGDyk (cyclic Arg-Gly-Asp-Tyr-Lys) heterodimer was created. It was then labeled with iodine 125 (I-125) via histidine in the cMBP and tyrosine in the cRGDyk. Both in vitro and in vivo tests were performed to evaluate the tumor-targeting effectiveness and receptor-binding properties of cMBP-click-cRGDyk.	A biodistribution research study found that at 4 h, ^125^I-cMBP-GGG-SC had the greatest T/B. On the other hand, at 1 and 4 h, static pin-hole pictures of ^125^I-cMBP-GGG-SC revealed a comparatively poor tumor uptake and high body background activity, with considerably greater pancreatic and renal activities throughout. To increase the targetability for an in vivo cancer model, cMBP-GGG-SC had to be modified by the heterodimerization of two ligands, one of which targeted c-Met and the other integrin.
Therapeutic efficacy of antiglioma mesenchymal extracellular matrix ^131^I-radiolabeled murine monoclonal antibody(mab) in a human glioma xenograft model [95]	The discovery of Mabs—especially those reacting with primary brain tumors but not with the normal brain—offers a possible way to target human malignant gliomas specifically with therapeutic medicines. It has been demonstrated that Mab 81C6, an IgG2b immunoglobulin, binds to human glioma cell lines, glioma xenografts in nude mice, and primary human gliomas, but not to the normal adult or fetal brain. Mab 81C6 specifies an epitope of the glioma-associated extracellular matrix protein tenascin.	In several animal models, tumor-associated Mabs have demonstrated therapeutic effects. However, Mabs alone have often only proven effective against tiny or freshly infected tumors, with the exception of p 185, an IgG2a Mab directed against a neuoncogene-associated transmembrane glycoprotein. Drug–antibody conjugates have also only demonstrated activity against freshly infected tumor cells or in vitro. In contrast, a number of models have demonstrated the effectiveness of radiolabeled Mabs against well-established malignancies.
Mesenchymal stem cells in glioblastoma therapy and progression: how one cell does it all [96]	One of the somatic stem cells that is extensively studied and used in experimental treatments for the regeneration of damaged tissues is the mesenchymal stem cell (MSC). Furthermore, MSCs could have anti-tumor qualities, as was recently suggested. Glioblastoma (GBM) is a grade IV tumor of the central nervous system that has an unfavorable prognosis with no effective treatment currently available. Many debates have arisen from experimental trials that used MSCs to treat GBM. It has been demonstrated that native MSCs have anti-GBM action through apoptosis induction, cell cycle regulation, and angiogenesis control.	The actual nature of the connections between GBM cells and endogenous MSCs remains unknown; nonetheless, it appears that both cell types undergo functional alterations as a result of reciprocal signaling processes. MSCs grown in vitro appear to have GBM inhibitory properties. Notwithstanding these observations, a number of preclinical investigations showed that MSCs might effectively limit the development of GBM. Moreover, a number of strategies have demonstrated effective MSC-based drug delivery for anticancer purposes, which is extremely promising for potential therapeutic uses. However, as animal research provides the majority of experimental data, caution must be used when extrapolating this information to human treatment.
Selective sodium iodide symporter (NIS) gene therapy of glioblastoma mediated by EGFR-targeted lipopolyplexes [97]	When post-functionalized with ligands to improve targeting, lipo-oligomers offer a potential new vehicle for delivering therapeutic genes, like the sodium iodide symporter (NIS), to particular tumors. NIS is an effective theranostic technique for therapeutic radionuclide application and diagnostic imaging because of its iodide-trapping action. Applications of ^131^I allow for cytoreduction, whereas ^124^I PET imaging permits non-invasive monitoring of the in vivo biodistribution of functional NIS expression. We employed EGFR-targeted polyplexes (GE11) in our experimental design, which were first described in vitro using ^125^I uptake experiments.	Based on dosimetric calculations, NIS imaging enables an accurate assessment of radiation dosage for radioablation of the specific tumor. When ^131^I is applied, radionuclide entrapment occurs inside NIS-positive cells, and beta decay causes cell death. The impact of ^131^I is further enhanced by the cross-fire effect, which causes nearby cells to also undergo cytotoxic death. Because of their natural NIS expression, the thyroid and salivary glands are primarily affected by off-target damage. The TSH dependence of NIS expression results in a downregulation of thyroidal iodide absorption following pretreatment with LT4. Thyroid hormone replacement therapy can be used to treat hypothyroidism if it still develops following treatment. Even in cases of advanced metastatic illness, radioiodide therapy has a proven track record of success in treating thyroid cancer.
